# Controlling illegal stimulants: a regulated market model

**DOI:** 10.1186/1477-7517-5-1

**Published:** 2008-01-23

**Authors:** Mark Haden

**Affiliations:** 1 Vancouver Coastal Health Authority, Pacific Spirit Community Health Centre, 2110 West 43^rd ^Ave, Vancouver, British Columbia, V6K 2E1, Canada

## Abstract

Prohibition of illegal drugs is a failed social policy and new models of regulation of these substances are needed. This paper explores a proposal for a post-prohibition, public health based model for the regulation of the most problematic drugs, the smokable and injectable stimulants. The literature on stimulant maintenance is explored. Seven foundational principles are suggested that could support this regulatory model of drug control that would reduce both health and social problems related to illegal stimulants. Some details of this model are examined and the paper concludes that drug policies need to be subject to research and based on evidence.

## Commentary

The global movement toward recognizing the failure of drug prohibition is growing. This is partly due to the emergent understanding that drug prohibition is the dominant driver behind the creation of a illegal market that spawns significant health and social pathologies, harmfully engages our youth, and makes impure illegal drugs widely available. In Canada the concept of a regulated market has been proposed as an alternative to drug prohibition [1-3] and this reflects the growing international movement [4-6]. One of the next steps toward evidence-based drug policies is to develop specific models of drug control for each of the different classifications of drugs. These models should be able to demonstrate that their implementation would produce less harm than the current prohibitionist model. Different types of drugs will need different models of control due to their widely differing pharmacological attributes. The smokeable and injectable stimulants have a wide range of potential harms and therefore pose a considerable challenge to those proposing new, public health based models of drug control. The goal of this paper is to address this challenge and explore a specific model that could be used in a post-prohibition paradigm to reduce the harms caused by these specific substances. This paper will explore some of what is known about the patterns of stimulant drug use and recommend a more optimal policy direction than the current prohibitionist model.

The mass media in Canada describe the use of crystal methamphetamine as a plague [7] or epidemic [8] and warns of marijuana laced with crystal methamphetamine even when no contaminated marijuana is seized by the police [9]. Poll reports indicate that the media exaggerate the prevalence of use. In schools, use of crystal methamphetamine is likely very limited, as only 4–5% of students report having ever used this drug [10]. This use was probably not by injection, as reports show that only between 0–1% of students have ever injected drugs [10,11]. Use of cocaine by students is also low, at about 5% [11]. These usage rates are in sharp contrast to marijuana use rates, as over half of British Columbia's 17 year olds report having used this drug [11]. The use patterns of injectable or smokable stimulants are limited to the most marginalized populations, such as street youth, where frequency of use is significant and may be increasing. In the street youth population, 67–71% have used amphetamines and 57% have used them more than 10 times [12]. In Canada use of crack cocaine follows a similar pattern where the use is restricted to marginalized populations [13] and this same pattern is seen in the United States as well [14].

If an intervention is to be successful at reducing the health and social problems associated with stimulant drugs, it must at a minimum, be able to alter the behaviour of individuals who inject and smoke these drugs. Drug law enforcement is currently the predominant response to the problems created by illegal drugs use, with Canadians spending approximately CA$2.3 billion on direct law enforcement costs and CA$1.1 billion on health costs [15]. Because our society continues to approach drug use as primarily a criminal problem rather than a health problem we suffer from the ineffectiveness of this approach [16-20]. We are also burdened by health and social problems which are the unanticipated outcomes of prohibition [1,21].

These increased health and social problems created by drug prohibition can be attributed to three likely causes. The first is that enforcement interventions themselves are directly responsible for the creation of harmful health and social impacts. This is due to the fact that enforcement activities create a disruption of health service provision to drug users and as a result there is increased risk-taking behaviours associated with infectious disease transmission and overdose [22]. Fast, surreptitious, back-alley injections are rarely safe and hygienic.

Secondly, drug prohibition prevents the exploration of the potential benefits of currently illegal drugs, which could be used in the treatment of drug users. For example initial research has suggested that cannabis can be useful in hepatitis C treatment as it improves patient retention and outcomes in programs designed to treat this disease [23,24].

The third and most important reason is that the enforcement paradigm prevents the establishment of a regulated system of drug control that could more effectively mitigate the harms associated with illegal drug use. The discussion of a regulated market for all currently illegal drugs has recently been legitimised in Canada with the release of the three reports, one federal [3] one provincial [25] and one from the City of Vancouver [2]. All of these reports are significant as they move beyond criticisms of drug prohibition and recommend the creation of a regulated market for currently illegal drugs. It is therefore timely to consider how a public health model of drug control could reduce many of the health and social problems associated with these substances. A regulated market system could control who could access drugs, the training that users receive, and the context in which these substances are consumed. These controls can be predicted to reduce many health and social pathologies that are currently associated with the use of illegal stimulants.

Heroin maintenance, as a treatment regimen for opioid dependence, has existed in some countries for many years [26,27] and while there are important lessons that have been learned from this experience [28], there are unique features to establishing a stimulant maintenance program. As a result, effective control of stimulants needs to be considered as a separate and distinct challenge. Formulating an effective model for stimulant control is important in the discussion of the prevention of diseases like HIV/AIDS and hepatitis C, as this class of drugs poses a significant challenge to public health officials due to the frequent injection of these drugs [29] and the sometimes erratic behaviour of those engaging in this high-risk behaviour. The challenge of creating a rational public health response that is both compassionate and effectively controls stimulant use is perhaps one of the more difficult tasks faced by advocates of drug policy reform. In order to do this, it is appropriate to examine the existing work that has been done on stimulant maintenance.

Dexamphetamine was prescribed to 63 intravenous amphetamine users by McBride and colleagues who observed significant improvements in both health and social functioning of these individuals [30]. A low dosage oral amphetamine project was conducted over three years by Fleming and Roberts, who reported that this program helped patients to cease or reduce injecting and other risk behaviours, and the authors also noted an increase in users presenting for treatment [31]. In a pilot randomized double blind controlled study, Shearer found that those who were given dexamphetamine reported a reduction in cocaine use, criminal behaviour, and cravings for cocaine [32]. Sustained-release d-amphetamine was used by Grabowski in his treatment of cocaine dependence for clients on methadone [33]. When amphetamine was prescribed to patients with schizophrenia and amphetamine dependence, Carnwath and colleagues observed a benefit [34]. A brief positive report is offered by Sherman who treated 13 patients for methamphetamine addiction by giving them stable dosages of dexamphetamine [35]. Prescribing amphetamines is not uncommon in the UK as Strang estimated there were 900–1000 patients receiving amphetamines for the treatment of addiction in England and Wales [36]. Bruce observes that the Department of Health Guidelines in London indicate that the prescription of dexamphetamine is at times, appropriate. He examines a variety of treatment approaches for amphetamine dependence and concludes that under some circumstances, prescription of amphetamines is an option that should be explored [37]. A review article by Mattick and Darke explored concerns and offered tentative optimism about stimulant maintenance [38]. The literature on stimulant maintenance is also reviewed by Fleming, who concluded that this is a valid and useful treatment process [39]. This was echoed by Alexander who also did a literature review and concluded that stimulant maintenance could be a successful, pragmatic innovation [40].

An examination of the literature above leads to the conclusion that giving dependent users controlled access to stimulants has the potential to reduce risky and illegal behaviours and therefore improve health and social functioning. These papers start to challenge the basic assumptions of drug prohibition, but they do so only by implication. They do not go far enough and deal directly with the actual problem of prohibition itself, which is the paradigm that is responsible for such high infection rates and has spawned so many other health and social pathologies [1,21]. The goal of this paper is to move forward by thinking out of the "prohibition box" and suggesting a specific public health model of stimulant control that would significantly reduce or eliminate the illegal market, and therefore considerably reduce both the health and social problems associated with these compounds. In order to do this, seven foundational principles need to be explored:

### Principle 1: The goal is to reduce harm

While the goal of reducing harm may sound self-evident, it has not always been clear that this is the primary policy objective. The 1998 United Nations General Assembly Special Session on Drugs urged member states to work towards the goal of "a drug-free world by 2008" [41]. This goal entrenches the enforcement approach, and has paradoxically increased both health and social harms and produced a illegal market that makes drugs widely available. What is needed is a public health model of drug control that is driven by evidence and clearly establishes pragmatic, realistic, achievable targets for the reduction of harms to individuals, families and all of society.

### Principle 2: Social Capital needs to be increased

The concept of social capital [42-44] should be foundational in the post prohibition paradigm. This concept correlates strong, healthy, supportive and multigenerational bonds between individuals in families, schools, and communities with many health, social and economic benefits [45,46]. To put this concept simply: relationships have value. Those who have abundant social networks and the reciprocities which flow from them are happier, healthier and wealthier. The opposite is also true as marginalized, alienated individuals who are disconnected from a variety of supports suffer significant harms that result in many health, social and economic impacts [47]. The lens of social capital elucidates how social networks impact drug use [48] and is especially useful in understanding youth drug use [49]. Students with strong connections to family, school and community are healthier and were less likely to smoke cigarettes and marijuana or drink alcohol [11]. It was observed that resilience could be improved in vulnerable youth by improving school and family connections [50]. Social capital is also a concept vital to the understanding of disease risk factors and infection control [51]. Increase in social capital is also important for those who are recovering from substance abuse [52-54]. This concept is also significant from a community perspective. Mheen observed the role of social capital in keeping drug dealers from infiltrating into neighbourhoods, as illegal markets tend to flourish in areas where poor social cohesion results in difficulty regulating nuisance and problematic behaviours [55]. Ford explored how a drug market becomes established due to poor social capital and suggested that a regulated drug trade would support the well being of drug users and minimize nuisance factors [56]. The issue of social capital is also observed in research that explores aboriginal ayahuasca and peyote rituals that promote social cohesion and have demonstrated individual and community benefit [57-59]. This exploration suggests that marginalized drug users, who are most at risk for the transmission of diseases, can be greatly benefited by the increase of social capital in their lives. If the goal is to reduce or eliminate risky behaviours, increasing their strong and supportive bonds will be crucial to this process.

### Principle 3: The culture of drug use needs to be understood and influenced

Effective control of infectious diseases and social problems associated with illegal drugs will only be realized when the culture of drug use is understood and influenced. Drug use patterns within communities need to be understood as cultural patterns in that they are governed by a wide variety of social normative behaviours and rituals. This perspective is helpful in rethinking risk and risk management [60,61]. The complex and richly rewarding study of cross-cultural drug use [62-64] is very helpful in the development of an effective post prohibition model of drug control. For a model to be effective in the control of stimulant use, it would have to engage the drug using community in developing a new drug using culture that works actively to reduce harms. This culture could positively influence how, where and when drugs are used, and specify acceptable behaviours for those who are using these substances. The effect of a positive change in the drug using culture was observed in Switzerland where the incidence of heroin use dropped as the use of heroin was reframed as a behaviour that required medical attention. The Swiss successfully shifted the image of heroin use and made it unattractive for young people [65] and it is notable that this drop in use occurred in spite of fact that the Swiss are criticized for their pragmatic drug policies. The confines of the prohibitionist framework discourage the implementation of interventions that would support the positive evolution of the drug culture. This is because interventions that could create positive new behaviour patterns and protective social norms are extremely difficult to implement within the prohibitionist model.

### Principle 4: The goal is to use the "least restrictive" intervention

The principle of "least restrictiveness" states that each drug should be controlled using methods that are as minimally restrictive as is possible given that the goal is to achieve specific health and social outcomes. There are both human rights and economic justifications for this principle as both respect for individual autonomy and cost savings are important. Adopting this principle requires achieving a balance between the potential harmfulness of a drug with the appropriate level of control. There is a significant range of preparations of stimulant drugs available that have different potentials for harm. On one end of this continuum is a weak oral solution (chewing coca leaves, and drinking coca leaf tea). In the middle is "snorting" of a more concentrated powder, and at the riskiest end is the smoking and injecting of more highly concentrated products. South American indigenous peoples have drunk coca tea and chewed the leaves for over 3000 years [66], often on a daily basis with no harms to the individuals, families or communities [67,68]. On the other end of the spectrum are the significant problems that marginalized individuals experience when these drugs are injected or smoked in chaotic use patterns. Across the whole spectrum, it can be observed that the concentration of the product and the method of taking the drug and the context combine to produce a wide range of possible harms. The "least restrictiveness principle" requires that substances with greater potential for harm, like injectable or smokable preparations of cocaine, be controlled with more restrictive mechanisms. Less harmful preparations such as coca tea, can be appropriately controlled with social norms and rituals and therefore need fewer and less restrictive administrative interventions.

### Principle 5: Prevention and treatment are vital

A vital aspect of a post prohibition model is the need for effective treatment and prevention programs. Only after prohibition ends can these programs flourish, as the effects of prohibition are in direct opposition to the goals of effective treatment and prevention. There are (at least) two reasons why this is true. Prohibition impairs the development of honest, factual prevention programs [69-72] and prohibition marginalizes, and alienates drug users and this produces many health and social consequences. This fact has been explored in many significant Canadian reports [2,73-76]. The City of Vancouver report; Preventing Harm From Psychoactive Substance Use [77], explored in detail how a regulated market for currently illegal drugs is a basic requirement if the city is going to significantly impact the drug problems its citizens so often experience. Looking at prevention and treatment programs through a post prohibition lens allows these concepts to be expanded to embrace the social determinants of health (e.g. housing, poverty, empowerment, community cohesiveness) that are foundational to a public health understanding of addiction. There is a connection between the prevention and treatment literature and the social capital research. Effective prevention and treatment programs could have as a goal to increase the social capital in the lives of the participants or community residents. Another reason why prohibition is detrimental to the provision of treatment and prevention programs is that prohibition of drugs is a very expensive process. When examined through the lens of the Federal Drug Strategy it is observed that enforcement costs absorb 73% of the budget, treatment receives 14% and prevention receives 3% [78]. In order to provide effective and responsive treatment and prevention programs they would have to receive adequate funding and as tax dollars are always scarce, this dramatic imbalance would need to be rectified.

### Principle 6: Learn the lessons from alcohol and tobacco

We need to reflect on the lessons learned from the regulation of alcohol and tobacco as we develop a post prohibition model of stimulant control. These products have historically been under-regulated [25,79] and it is important to not repeat these errors. When a product is allowed to be branded, the battle to control the advertising of it is relentless [80]. Only in a tightly controlled government regulated system could the presentation of a product be consistently uniform and deliberately unattractive [81].

### Principle 7: Changes need to occur incrementally

Drug policy needs to change incrementally as policy makers need time to gather evidence and change direction based on collected data. The public also needs time in order to be reassured that the change is beneficial and that we are moving toward the goals of reducing the health and social problems associated with illegal drugs. Health harms are related to the spread of disease and premature death. Social harms are mostly related to the functioning of the illegal market, which is responsible for the violence, crime, corruption, uncontrolled availability of drugs, and the engagement of youth into the drug scene. Data collection on all of these issues at each incremental step will be important for policy makers and the public. Response to this data is vital as policy can be stabilized when the illegal market is significantly decreased or eliminated and drug users are in safe environments.

There are a variety of increments that can be used. Initially, policies might allow only drug-dependent adults to legally access substances. Then, as health and social improvements are demonstrated in this population, adults who use drugs problematically could be included. If positive data continues to emerge and the illegal market is still functional, the next step would be to include drug using adults who have been trained in ways of reducing potential harms. Increments can also be based on drug price. Substances can initially be priced at 80% of the illegal market price, and if the illegal drugs are still widely available, the price can slowly be reduced until the illegal market either ceases to function or is substantially reduced to the point where it produces minimal harms. Increments could also be based on "order-delivery delay time". Initially there could be a delay of 48 hours between customer order and delivery of the product. This would reduce the chance of uncontrolled sequential use. This delay time could be reduced as the size of the illegal market was monitored. Delay time could be stabilized at the point at which the illegal market is reduced to a size where it inflicts minimal health and social damage. Another incremental change could be based on geography, as one neighbourhood could be selected for a pilot study and as benefit is proven the area could then be expanded. Type of drug preparation that is available, could also be incrementally changed. Initially only weak oral solutions could be purchased, and in response to the evidence, stronger preparations could be made available. There can also be a slow incremental transition from administrative to social controls as our society becomes more sophisticated at influencing the social norms that control drug use patterns and drug user groups mature and evolve to take more responsibility for the behaviour of their members.

When the above seven principles are combined in a public health model, we can make specific drug control policy recommendations, and we can replace the prohibition of stimulants with a more rational policy that would significantly reduce the health and social problems associated with stimulant drugs.

While there are many types of controls that could be used [4,5,82] this paper will explore only a few of these that show promise for specifically reducing the health and social problems associated with injectable and smokeable stimulants. The following controls are based on the above principles:

#### Age

These substances should not be sold to youth under the age of 19, as we have learned from our experience with alcohol and tobacco that we can reduce access in the youth population with age controls [83].

#### Required training prior to purchase

Consumers of more concentrated products need to be enrolled in a training program that provides them with information about how to reduce or avoid harm. This training would have a prevention and treatment focus, and would cover both prevention of harms and access to services, for those who need treatment for substance dependence.

#### Required consumption of drugs at a safe, health promoting facility

As concentrated injectable and smokeable stimulants need to be removed from the illegal market, take out dosages should not be available. These substances will need to be consumed at a supervised consumption site where they are dispensed. The behaviour of individuals using smokeable and injectable stimulants can become erratic, therefore consumers need to be observed to provide assistance or intervention. These health facilities need to be clean and engaging. Health care workers and peers could monitor consumers and, if indicated, provide assistance and referrals to other components of the addiction treatment system. InSite, Vancouver's supervised injection facility provides assistance and referrals to other treatment services [84] and this is consistent with the experience in other countries. For example the Swiss heroin prescription trial was successful in engagement of drug users as 22% of the clients went on to become involved in abstinence based services [85].

#### Voluntary and involuntary "cut-offs" should be available

As problematic binge use of stimulant drugs is observed in some individuals, "cut-offs" need to be established. The "least restrictive" principle dictates that initially "cut offs" should be voluntary where the consumption room staff negotiate in advance with the customer and plan for consumption levels. If this is not an adequate intervention and problematic behaviours are observed, then involuntary "cut-offs" would be needed to assist those who are experiencing harm from these substances.

#### Membership in a drug users group would be required

One of the goals of an effective drug policy will be to both increase social capital and directly influence the culture of the drug using community. This can be achieved through sharing of responsibility where drug user groups would be established and then held partially responsible for the behaviour of their members. These drug user groups will need to be legitimate, secure and adequately funded. They would function as licensed bodies, and would be required to provide peer training. Group members would assist in running consumption facilities and work with peer pressure to reduce harmful behaviours. These groups would ideally be comprised of individuals from many sectors of society and would be able to provide public education with the goal of changing the social norms that influence all drug users. As the intention is to increase social capital within this community, users will need to participate in a legitimate and meaningful way [86]. In return for this legitimacy, accountability for specific outcomes (e.g. no criminality for members) would be required.

The above model is designed to be applied to smokable or injectable stimulants, which have significant potential for harms [87]. Controls for a weak oral solution of cocaine and amphetamines should, as per the "least restrictiveness" principle, be significantly less than for the concentrated product as the potential for harm is much lower. Controls similar to those that exist in Canada for tobacco and alcohol would be sufficient for these products, with the significant exception of product branding and marketing, which would contribute to the increased consumption of these drugs. Therefore items like coca tea would be available to any adult, in plain packaging, with information for the consumer included. The current look of prescription drugs would be the desired appearance. Research data would need to be gathered from this experiment, and increases in the number and types of controls would have to be implemented if there was any evidence of increased health or social harms.

As the new post prohibition model would need to be based on evidence, new questions will need to be asked; for example "To what extent can a dependent user of crack cocaine be persuaded to substitute a less harmful weak oral solution?". There are clues that this is possible in some users as it has been observed that chewing coca leaves can improve the lives of coca paste smokers [88]. This positive transition was also observed when dependant coca paste smokers used coca tea and had fewer relapses, reduced cravings and longer periods of abstinence with no medically adverse effects [89].

The author of this paper concludes that health and social problems associated with currently illegal drugs cannot be significantly controlled unless drug prohibition is fundamentally challenged. While harm reduction programs like needle exchanges and supervised injection sites can reduce health and social problems, they are insufficient as they do not target the illegal market that supplies these substances and creates many of the problems that are associated with illegal drugs. If the above seven principles were combined to produce a system of tightly regulated access to concentrated stimulants and easier access to weak oral solutions, incidence of diseases like HIV/AIDS and hepatitis C, could be significantly reduced. As this system would significantly reduce the illegal market and associated criminality this model can also be predicted to reduce many social harms.

Currently the blunt instrument of prohibition prevents us from approaching drug use in our society with the level of finesse that is required to fine tune a new public health system that is responsive to evidence and evolves as new data emerges. The public has had many years of exposure to "drug war" messages that often directly and knowingly contradict the research evidence [90,91], and usually evoke a fear reaction. Changes in public perception will require more policy research, and the creation of prevention and education programs that are both factual and honest. These programs will need to explore both the realities of substance use and also examine the evidence that details the ineffectiveness and harms created by drug prohibition. Hopefully we live in a society that has enough collective wisdom to mature beyond our current fear-based approaches to allow the evolution of a public health model that is guided instead by evidence and compassion.
